# A voxel‐wise uncertainty‐guided framework for glioma segmentation using spherical projection‐based U‐Net and localized refinement

**DOI:** 10.1002/mp.70360

**Published:** 2026-02-27

**Authors:** Zhenyu Yang, Chen Yang, Rihui Zhang, Chunhao Wang, Minbin Chen, Fang‐Fang Yin

**Affiliations:** ^1^ Medical Physics Graduate Program Duke Kunshan University Kunshan Jiangsu China; ^2^ Jiangsu Provincial University Key (Construction) Laboratory for Smart Diagnosis and Treatment of Lung Cancer Kunshan Jiangsu China; ^3^ Department of Radiotherapy and Oncology The First People's Hospital of Kunshan Kunshan Jiangsu China; ^4^ Department of Radiation Oncology Duke University Durham North Carolina USA

**Keywords:** deep learning, glioma segmentation, spherical projection, uncertainty quantification

## Abstract

**Background:**

Accurate segmentation of glioma subregions from multi‐parametric MRI (MP‐MRI) is critical for clinical management but remains challenging due to tumor heterogeneity and ambiguous tissue boundaries.

**Purpose:**

This study proposes an uncertainty‐guided hybrid segmentation framework that integrates spherical projection‐based 2D modeling with localized 3D refinement to improve segmentation fidelity.

**Methods:**

The framework was validated on the BraTS 2020 dataset (*N* = 369). First, a 2D nnU‐Net with spherical projection deformation was employed to generate initial slice‐wise predictions. Crucially, prediction variance across multiple spherical projections was utilized to quantify voxel‐level uncertainty, highlighting regions of low model confidence. A kernel‐based sliding window algorithm then spatially localized 3D subvolumes with high cumulative uncertainty. These targeted regions were subsequently fed into a dedicated 3D nnU‐Net for volumetric refinement. Finally, the global 2D predictions and local 3D refinements were adaptively fused using weights optimized via Particle Swarm Optimization. The proposed method was implemented to segment the enhancing tumor (ET), tumor core (TC) and whole tumor (WT). Performance was evaluated against standalone 2D and 3D nnU‐Net baselines using the Dice Similarity Coefficient (DSC), HD95, sensitivity, and specificity.

**Results:**

The proposed method significantly outperformed 2D and 3D baselines across ET, TC, and WT targets. Notably, it achieved a DSC of 0.8124 for ET (vs. 0.7527 for 2D and 0.7530 for 3D), 0.7499 for TC (vs. 0.7002 for 2D and 0.7027 for 3D), 0.9055 for WT (vs. 0.8989 for 2D and 0.9038 for 3D) and demonstrated consistent gains in HD95 and sensitivity. Quantitative metrics and visualizations confirmed improved spatial coherence and boundary preservation in structurally complex regions.

**Conclusion:**

By utilizing interpretable uncertainty maps as a spatial attention mechanism, this approach dynamically allocates computational resources to anatomically ambiguous regions. The resulting hybrid framework successfully combines 2D efficiency with 3D contextual accuracy, offering a robust solution for automated glioma segmentation.

## 1 INTRODUCTION

Gliomas represent one of the most aggressive and prevalent forms of primary malignant brain tumors in adults, originating from glial cells within the central nervous system.^1^, ^2^ Glioblastoma multiforme (GBM) is one of its aggressive subtypes with heterogeneous morphology, highly invasive growth patterns, and significant resistance to standard therapeutic interventions.^1^, ^2^ In the clinic, accurate delineation and segmentation of glioma subregions are critically important for precise diagnosis, effective therapeutic planning, and reliable longitudinal monitoring. For example, reliable segmentation enables neurosurgeons to optimize the extent of tumor resection, assists radiation oncologists in accurately defining radiotherapy target volumes, and provides clinicians with objective metrics for assessing treatment response and disease progression.^3^, ^4^, ^5^ Currently, multi‐parametric magnetic resonance imaging (MP‐MRI) has been widely accepted as the clinical standard imaging modality for glioma diagnosis and evaluation. Due to its superior soft‐tissue contrast, MP‐MRI effectively characterizes tumor morphology, cellular composition, and peritumoral edema. Nevertheless, accurate segmentation of gliomas from MP‐MRI data remains challenging.^3^, ^4^, ^5^ Traditional manual segmentation methods, despite their widespread clinical use, are labor‐intensive, prone to substantial inter‐ and intra‐observer variability, and difficult to implement for routine or large‐scale clinical deployment.^6^, ^7^, ^8^

Recent advances in artificial intelligence have led to the widespread development of deep learning (DL)‐based approaches for automated glioma segmentation. Among them, convolutional neural networks (CNNs), particularly U‐Net and its variants, have achieved strong performance on benchmark datasets such as BraTS by leveraging multi‐modal MP‐MRI inputs.^6^, ^7^, ^8^ Most of these models, including recent large‐scale generalist frameworks such as the Segment Anything Model (SAM), perform segmentation in a 2D slice‐by‐slice manner.^9^, ^10^ The final volumetric segmentation is typically reconstructed by stacking predictions across slices. This design simplifies model training and enables deployment with limited GPU memory, but it fails to capture spatial continuity along the axial (*z*‐axis) direction. As a result, such models are prone to discontinuities between adjacent slices, misalignment at tumor boundaries, and anatomically implausible segmentations.^11^ These limitations become particularly pronounced in glioma subregions with subtle contrast differences or irregular shapes.^3^, ^4^, ^12^, ^13^, ^14^ For example, the non‐enhancing tumor (ET) core often exhibits low signal intensity on both T1 and T1ce, making it difficult to delineate consistently in 2D. Similarly, peritumoral edema seen on FLAIR may span several slices with gradual intensity transitions, which are easily missed without 3D context.^15^, ^16^ Although 3D CNNs such as V‐Net and 3D U‐Net have been proposed to address these issues by directly modeling volumetric context,^17^, ^18^, ^19^ they introduce a high computational burden and are sensitive to voxel anisotropy, particularly in clinical scans where slice thickness is coarser than in‐plane resolution. In addition, the relatively small tumor‐to‐brain volume ratio further complicates 3D learning due to severe class imbalance and limited availability of high‐quality 3D annotations. These practical constraints continue to limit the robustness, efficiency, and clinical applicability of existing DL‐based glioma segmentation solutions.

To address the limitations of current segmentation methods, recent research has increasingly focused on uncertainty quantification as a strategy to improve both algorithmic reliability and human‐machine interaction.^12^, ^13^, ^20^, ^21^, ^22^, ^23^, ^24^ Unlike traditional segmentation models that produce deterministic binary masks, uncertainty‐aware frameworks generate voxel‐wise confidence scores, offering more informative outputs that highlight regions of potential error. These voxel‐level uncertainty maps serve as indicators of prediction confidence and can guide clinicians to focus their review on ambiguous or high‐risk areas, thus improving both efficiency and reliability in clinical workflows.^22^ Technically, uncertainty in deep learning is broadly categorized into two types: epistemic and aleatoric. Epistemic uncertainty reflects uncertainty in the model parameters due to insufficient or biased training data and can be estimated through Bayesian neural networks^25^ or Monte Carlo (MC) dropout.^26^ In MC dropout, multiple forward passes with randomly dropped neurons are used to simulate a posterior distribution over model predictions. Aleatoric uncertainty, in contrast, arises from inherent noise in the input data, such as poor tissue contrast or motion artifacts, and is often estimated using test‐time augmentation (TTA).^27^ TTA introduces controlled perturbations to the input, such as flipping, rotation, or intensity scaling, and quantifies the variation in output predictions across augmented samples.

Building on these concepts, our study recently introduced a U‐Net architecture with spherical projection‐based image deformation to quantify segmentation uncertainty in brain tumor MRI.^12^, ^13^ Unlike methods that estimate uncertainty from feature distributions or latent activations, this technique operates directly in the input domain. Each 2D axial slice is nonlinearly projected onto a set of spherical surfaces to amplify local geometric features while preserving global structure. The projected spherical images are independently segmented by a shared 2D nnU‐Net, and the variance across the resulting segmentations is used to derive uncertainty. Empirically, the produced uncertainty maps consistently highlight clinically relevant failure‐prone regions, such as subtle tumor margins, necrotic cores with heterogeneous signal, and peritumoral edema.^12^, ^13^

Extending this work, the present study proposes a novel uncertainty‐guided hybrid segmentation framework designed to enhance the volumetric delineation of glioma subregions. To explicitly distinguish our contributions from prior approaches, this study introduces three key methodological innovations: First, unlike conventional frameworks that treat uncertainty quantification primarily as a post hoc evaluation metric or diagnostic visualization, we repurpose it as an active spatial attention mechanism. This establishes a functional coupling where uncertainty directly guides boundary correction, allowing the model to dynamically identify and prioritize “hard examples” for refinement. Second, we integrate spherical projection not merely for data augmentation, but as a test‐time ensemble generator. This approach provides robust entropy‐based uncertainty estimation without the substantial computational cost associated with training multiple deep networks (e.g., Deep Ensembles).^28^ Third, the kernel‐based refinement strategy bridges the gap between 2D efficiency and 3D contextual accuracy. By limiting 3D processing to anatomically ambiguous regions, the framework achieves high‐resolution volumetric segmentation where it matters most, while minimizing computational redundancy in high‐confidence areas. Collectively, this adaptive pipeline represents a methodological shift from static segmentation architectures toward dynamic, interpretable workflows that offer improved robustness and clinical utility.

## 2 MATERIALS AND METHODS

### 2.1 Patient data

**FIGURE 1:**
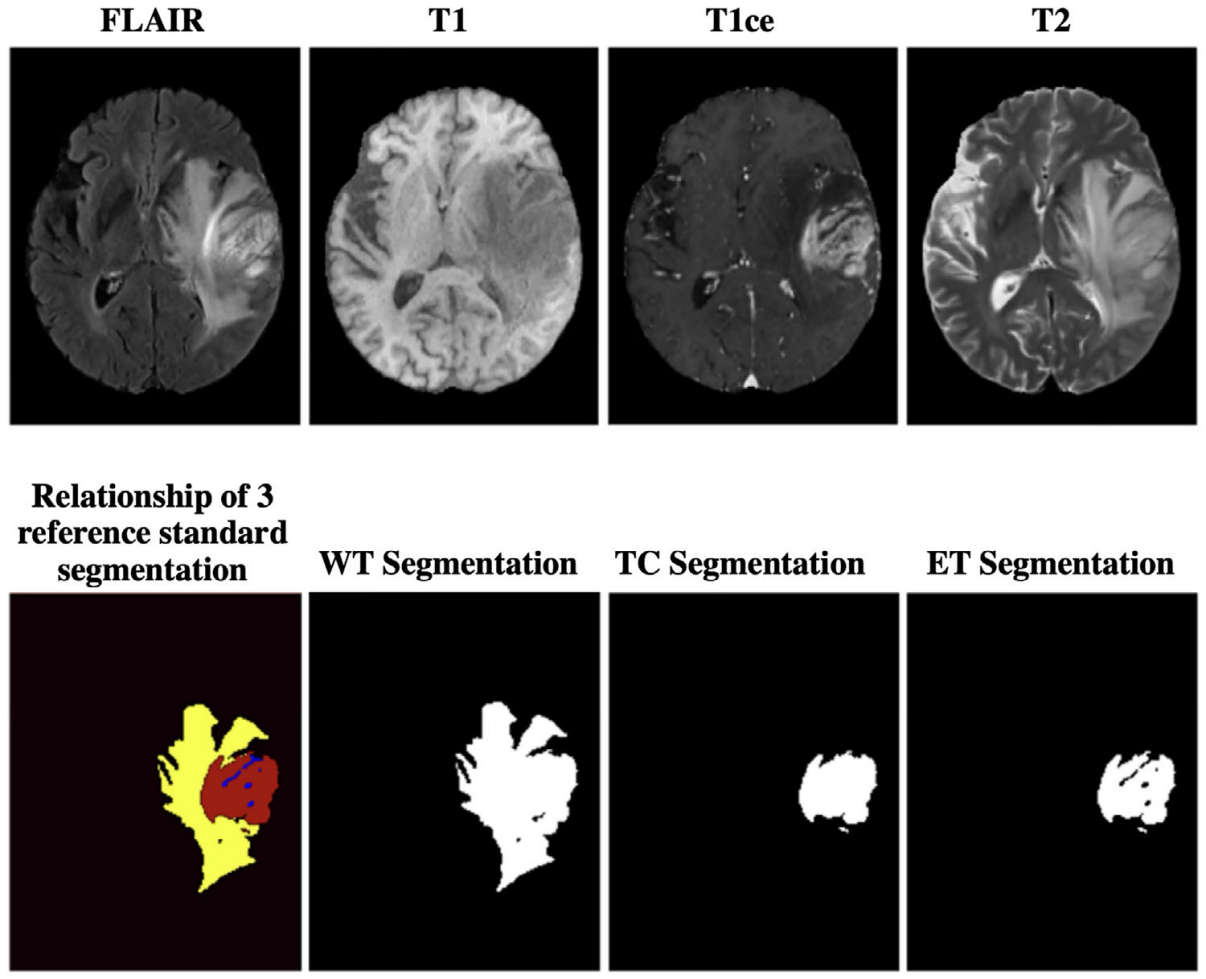
An example of 4 MR images (i.e., FLAIR, T1, T1ce, and T2) and the corresponding 3 reference standard segmentations (i.e., ET, TC, and WT) from the BraTS challenge 2020 dataset.

The study employed a total of 369 glioma patients from the Brain Tumor Segmentation Challenge 2020 (BraTS 2020) dataset.^29^, ^30^, ^31^ For each patient, 4 MR images were acquired as an MP‐MRI protocol, including T1‐weighted, T1‐weighted contrast‐enhanced (T1ce), T2‐weighted, and fluid‐attenuated inversion recovery (FLAIR). All MR images were preprocessed following the BraTS standard pipeline: skull‐stripping, rigid registration to a common anatomical space, and isotropic resampling to a voxel resolution of 1 × 1 × 1 mm^3^. To ensure consistent spatial dimensions, all volumes were zero‐padded to a matrix size of 256×256×160. The reference standard segmentation includes three non‐overlapping regions: Gadolinium (Gd)‐enhancing tumor (ET), peritumoral edema (ED), and non‐ET core (NCR/NET). Following the BraTS 2020 studies, another three overlapping reference standard segmentation targets were employed to evaluate the segmentation algorithms: ET, TC, (the combination of ET and NCR/NET), and whole tumor (WT, the combination of ET, NCR/NET, and ED).^29^, ^30^, ^31^ All segmentations were identified by 1–4 raters following a shared protocol, and the resulting labels were approved by experienced neuroradiologists.^29^, ^30^, ^31^ Figure 1 shows an example of the relationship between ET, TC, and WT.

### 2.2 Uncertainty‐guided segmentation model design

**FIGURE 2:**
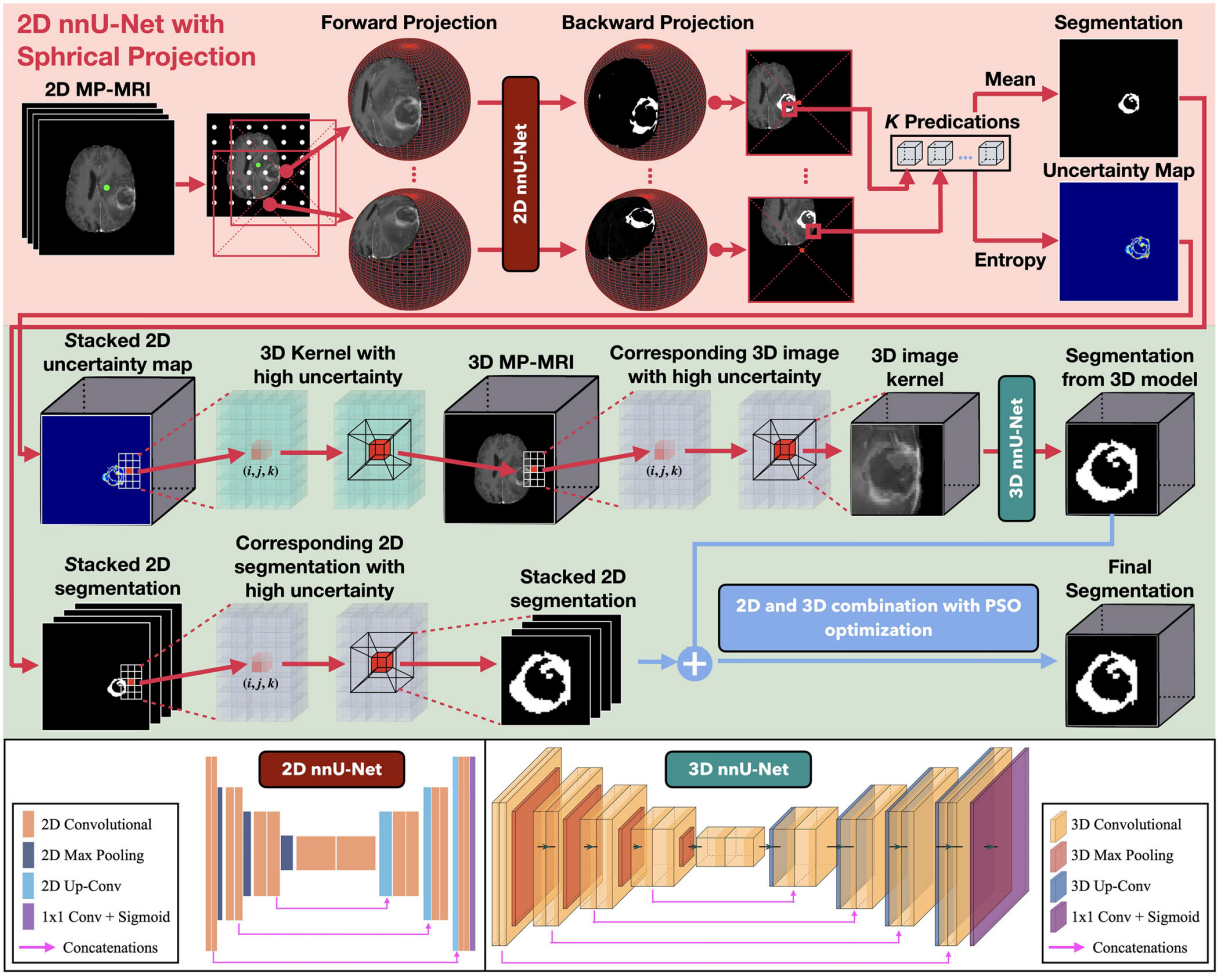
Workflow of the proposed uncertainty‐guided hybrid segmentation framework. The top panel (red) illustrates uncertainty quantification via spherical projection, where each 2D MP‐MRI slice undergoes 1024 spherical transformations and is segmented by a 2D nnU‐Net to generate a mean prediction and voxel‐wise uncertainty map. The middle panel (green) depicts kernel‐based extraction of high‐uncertainty subvolumes, in which a sliding 3D window identifies regions with maximal cumulative uncertainty for targeted refinement. The bottom panel shows uncertainty‐guided 3D segmentation and voxel‐wise fusion: selected subvolumes are processed by a 3D nnU‐Net, and the refined 3D predictions are combined with the global 2D segmentation using PSO‐optimized weighting to produce the final segmentation output. Architectural diagrams of the 2D and 3D nnU‐Net backbones are included for reference.

The architecture of the proposed uncertainty‐guided hybrid segmentation framework is systematically illustrated in Figure 2. The workflow integrated three sequential stages: (1) *Uncertainty Quantification*: a 2D nnU‐Net utilizing spherical projections generated preliminary segmentation masks and, crucially, produced voxel‐wise uncertainty maps from MP‐MRI to quantify confidence and identify regions of anatomical ambiguity; (2) *ROI Localization*: these uncertainty signals were aggregated using a sliding window algorithm to spatially locate specific 3D subvolumes requiring further refinement; and (3) *Uncertainty‐Guided Segmentation Refinement*: the identified high‐uncertainty regions were extracted from the original MP‐MRI data and processed by a dedicated 3D nnU‐Net. The final volume segmentation was achieved by fusing global 2D predictions with local 3D refinement results using an adaptive weighting strategy.

#### 2.2.1 Uncertainty quantification

The segmentation uncertainty quantifies the predictive confidence for each voxel's class assignment within the target segmentation region. Following our previous work,^12^, ^13^ the voxel‐level glioma segmentation uncertainty can be estimated using a geometric image transformation method inspired by spherical image processing.^32^ As depicted in the top panel (red) of Figure 2, each 2D axial MR image slice was initially projected onto a series of virtual spherical surfaces, each associated with a unique projection origin. The spherical projection process introduces structured, nonuniform distortions across the image: anatomical details proximate to the projection origin are magnified, while the overall field‐of‐view and contextual integrity are largely preserved. To achieve comprehensive coverage and diverse transformations, we strategically placed 1024 projection origins throughout each 256 × 256‐pixel MR image slice. These origins were uniformly distributed at an 8‐pixel interval in both dimensions, resulting in 1024 unique spherical projections per 2D slice.

For each 2D axial slice of an MRI scan, a consistent set of 1024 spherical projections (corresponding to 1024 origin points) was applied across all four MRI modalities—FLAIR, T1, T1ce, and T2. These projected images were concatenated into a single 4‐channel tensor (i.e., 4×h×w), serving as the input for a 2D nnU‐Net model.^33^, ^34^, ^35^, ^36^ The nnU‐Net is widely recognized as a state‐of‐the‐art backbone for medical image segmentation due to its robust performance and adaptive capabilities.^33^, ^35^ Its architecture is characterized by a symmetric encoder–decoder structure designed to capture both local spatial features and global contextual information. The encoder comprises multiple downsampling blocks, each consisting of two convolutional layers followed by instance normalization and Leaky ReLU activation, which effectively extract hierarchical features while progressively reducing spatial resolution. The decoder mirrors this structure with a series of upsampling blocks, employing transposed convolutions and skip connections that bridge corresponding encoder layers to facilitate multi‐scale feature integration. A final sigmoid activation layer outputs the probability (range [0,1]) of each pixel (within the spherical surface) belonging to a specific target class (e.g., ET, TC, or WT). Unlike other U‐Net variants, nnU‐Net's key advantage lies in its automatic configuration of architectural parameters such as input patch size, network depth, normalization type, and data augmentation strategies based on input data analysis.^33^, ^35^ This self‐configuring capability significantly enhances its robustness across diverse imaging modalities and anatomical targets.

For each 2D axial MRI slice, the nnU‐Net provides 1024 independent segmentation prediction probabilities (range [0,1]), corresponding to the 1024 spherical projected 2D MP‐MRI images. An initial 2D segmentation mask $P_{2D}\left(i,\;j\right)$ was derived by averaging these 1024 predictions and binarized when a mean probability value exceeds 0.5. Segmentation uncertainty $U_{2D}\left(i,j\right)$ was subsequently quantified by assessing the variability among these projected probabilities using Shannon entropy.^37^ Each continuous prediction value (range [0,1]) was first linearly discretized into 100 bins. The uncertainty $U_{2D}\left(i,j\right)$ was then calculated as:

(1) $$U_{2D}\;\left(i,j\right)=-\sum_{t\;=1}^{T}f_{2D}^{\left(t\right)}\left(i,j\right)\cdot \ln\left(f_{2D}^{\left(t\right)}\left(i,j\right)\right),$$

where $f_{2D}^{\left(t\right)}\left(i,j\right)$ denotes the empirical frequency of the t‐th discretized bin across all projections for the pixel (i,j), and T denotes the total number of unique nonzero bins in the pixel's uncertainty distribution. As such, the entropy captures prediction dispersion: low entropy indicates high consistency among model predictions under 1024 spherical projections (i.e., high confidence), while high entropy reflects significant disagreement (i.e., low confidence). By processing the entire 3D MP‐MRI volume slice‐by‐slice, this 2D nnU‐Net generates both the binarized segmentation mask $P_{2D}\left(i,\;j,k\right)$ and a corresponding voxel‐wise uncertainty map $U_{2D}\left(i,j,k\right)$ for the entire volume. Note that the calculated voxel‐wise uncertainty (Equation 1) reflects the variability of the segmentation prediction given the full multi‐parametric MRI (MP‐MRI) context, rather than deriving from individual MRI modalities in isolation. A more detailed description and implementation specification of this spherical projection segmentation model can be found in Reference.^12^, ^13^

#### 2.2.2 Kernel‐based identification

A kernel‐based iterative search algorithm was subsequently developed to systematically identify 3D subvolumes exhibiting high segmentation uncertainty. As illustrated in the middle panel (green) of Figure 2, a cubic sliding window kernel with a fixed dimension of $d\times d\times d\;mm^{3}$ was traversed across the entire uncertainty map $U_{2D}\left(i,j,k\right)$ in a dense grid. At each discrete position, the cumulative uncertainty within the enclosed region was computed as the sum of its constituent voxel‐wise uncertainty values. The region yielding the highest cumulative uncertainty was initially selected as the primary candidate for further refinement. Subsequent candidate kernels were identified using the same procedure, with an additional constraint on permissible volumetric overlap between newly selected and previously chosen kernels. If a newly selected kernel exhibited a volumetric overlap with any previously chosen kernels exceeding this predefined constraint, it was discarded; otherwise, it was considered as a valid candidate for refinement. This iterative selection process continued until the cumulative uncertainty in all remaining unselected kernels approached a predefined minimum threshold, effectively identifying all significant high‐uncertainty regions.

**FIGURE 3:**
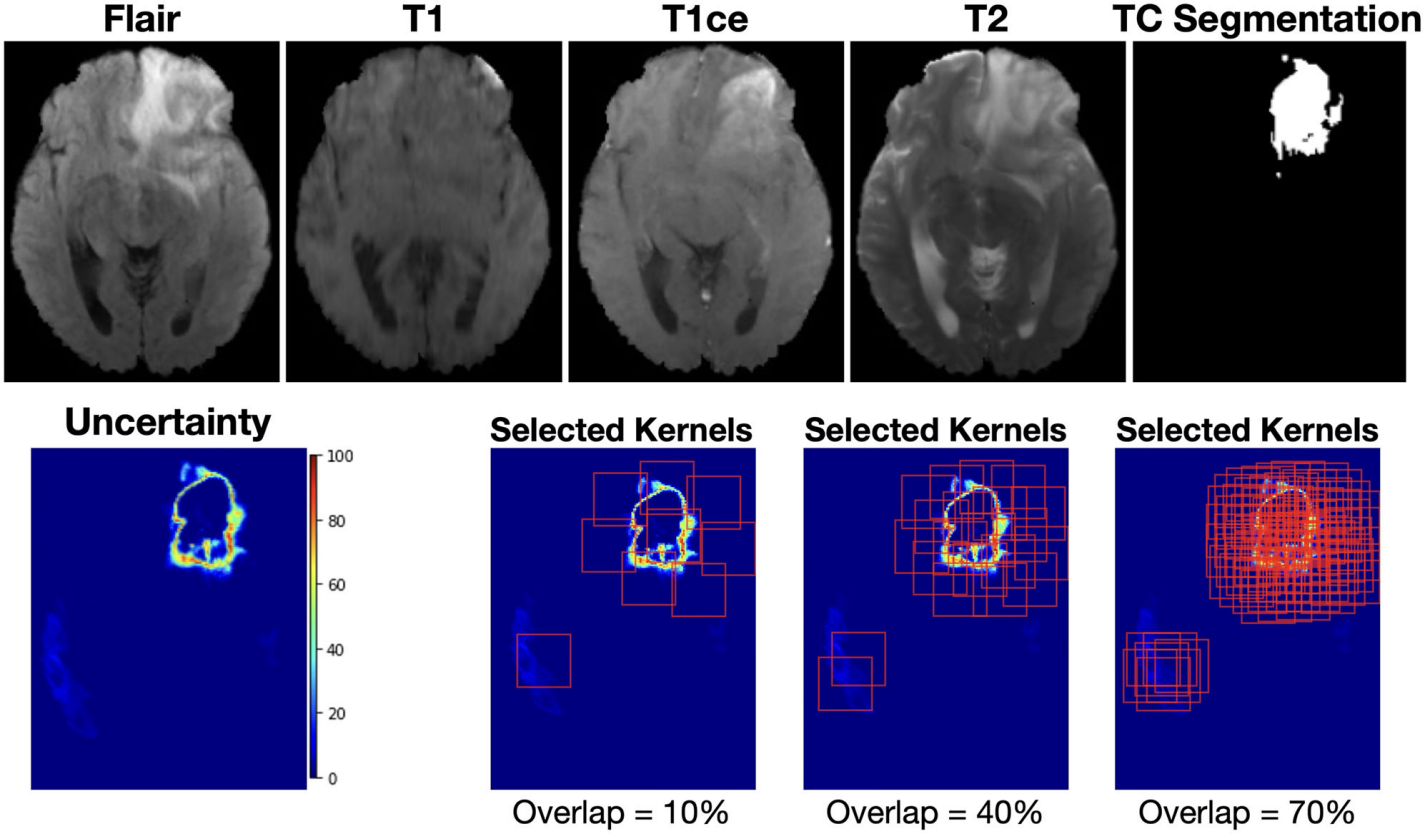
Illustration of uncertainty‐guided kernel selection with varying overlap ratios. Top row shows representative axial MP‐MRI slices (FLAIR, T1, T1ce, and T2) and reference standard TC segmentation. The bottom row displays the voxel‐wise uncertainty map generated by U‐Net with spherical projection deformation and selected refinement kernels (red boxes) overlaid on the uncertainty map using three different overlap settings: 10%, 40%, and 70%.

The kernel size (d) was treated as a critical hyperparameter influencing the granularity of uncertainty localization. A smaller kernel risks overfitting to localized noise or minor uncertainties, whereas an excessively large kernel may incorporate non‐informative background, thereby compromising the precise localization of uncertainty. Based on a statistical analysis of the training cohort (median tumor dimensions: ∼42–53 mm for ET/TC and ∼83 mm for WT), we empirically selected compact kernel sizes d=32mm for ET and TC, and d=64mm for WT. These dimensions were rounded down to the nearest power‐of‐2 for ease of calculation and computational efficiency (details can be found in *Figure* S–1 of the *Supplementary Materials*). This compact strategy prioritizes high‐frequency local boundary features, relying on the sliding window mechanism to seamlessly cover larger anatomical extents via patch aggregation. The volumetric overlap constraint is another crucial hyperparameter. As conceptually demonstrated in Figure 3, excessive overlap (e.g., 70%) between selected kernels results in redundant subvolumes with highly similar anatomical content. Incorporating such highly overlapping kernels for subsequent refinement could potentially increase computational burden and lead to overfitting. Conversely, an insufficient overlap ratio (e.g., 10%) risks under‐covering critical high‐uncertainty regions, leading to incomplete refinement. In this study, we empirically set the volumetric overlap constraint to 40% of the entire 3D kernel volume ($d\times d\times d\;mm^{3}$).

This iterative search algorithm effectively ranks potential kernel regions by cumulative uncertainty, prioritizing areas of higher uncertainty while ensuring diversity by rejecting redundant, highly overlapping candidates. For each patient, the selected 3D image kernels (for all 4 MRI modalities), along with their corresponding 3D reference standard segmentation (for three respective tumor subregions), were extracted for subsequent refinement.

#### 2.2.3 Uncertainty‐guided segmentation refinement

The kernel‐based identification step serves as a spatial attention mechanism to guide volumetric refinement. For each identified high‐uncertainty subvolume, the corresponding 4‐channel MP‐MRI data (comprising FLAIR, T1, T1ce, and T2 sequences) was extracted from the original patient scan. Crucially, these cropped MRI subvolumes—rather than the uncertainty maps themselves—served as the input to a dedicated 3D nnU‐Net. This input consists of 4D tensors with dimensions of 4×d×d×d. This design effectively implements a two‐step strategy: by training exclusively on regions where the 2D model lacked confidence (i.e., ambiguous boundaries or heterogeneous cores), the 3D network concentrates its learning capacity on resolving complex local topologies using full volumetric context. Structurally, this 3D nnU‐Net shares an identical design with the 2D nnU‐Net described previously, with direct replacement of 2D operations (convolution, pooling, upsampling) with their 3D counterparts. Consequently, for a given voxel (i,j,k) in the original MR image, one or more refined 3D segmentation probability maps, denoted as $P_{3D_{local}}\left(i,j,k\right)$​, are obtained from the 3D nnU‐Net (allowing for overlapping predictions from distinct kernels).

In parallel, the original 2D segmentation prediction from the U‐Net with spherical projection deformation, $P_{2D}\left(i,j,k\right)$, is also available for the same spatial location. These two predictions are then integrated using a voxel‐wise fusion strategy designed to optimally combine (1) the global contextual understanding from the 2D nnU‐Net with (2) the precise local refinement from the 3D nnU‐Net. This fusion is governed by a sigmoid‐weighted linear combination for each spatial location within the MP‐MRI volume, expressed as:

(2) $$\begin{array}{cc} & P_{\mathrm{fused}}\;\left(i,j,k\right)\\ & \;=\sigma \left(w_{2D}\cdot P_{2D}\left(i,j,k\right)+w_{3D}\cdot P_{3D_{\mathrm{composite}}}\left(i,j,k\right)+b\right), \end{array}$$

Here, $p_{\mathrm{fused}}$ is the final fused segmentation probability, $w_{2D}$ and $w_{3D}$ are weighting coefficients for the 2D and 3D models, respectively, b is a bias term, and σ denotes the sigmoid activation function that transforms the combined linear output into a probability in the range [0,1]. The fusion logic adapts dynamically based on 3D kernel coverage:

1. For voxels not covered by any extracted 3D high‐uncertainty region, no 3D prediction is available. In these instances, the original 2D nnU‐Net prediction is retained as the final segmentation output for that voxel.
2. For voxels covered by exactly one 3D kernel, $P_{3D_{\mathrm{composite}}}\left(i,j,k\right)$​ equals $P_{3D_{\mathrm{local}}}\left(i,j,k\right)$ prediction from that specific kernel, and fusion Equation (2) is applied.
3. In regions with multiple overlapping 3D kernels, multiple $P_{3D_{\mathrm{local}}}\left(i,j,k\right)$ predictions are available for a given voxel. These are averaged to form a composite 3D probability: $P_{3D_{\mathrm{composite}}}\;\left(i,j,k\right)=\frac{1}{N}\;\sum_{n=1}^{N}P_{3D_{\mathrm{local},\;n}}\left(i,j,k\right)$, where N is the number of overlapping kernels contributing predictions to voxel (i,j,k). Subsequently, fusion Equation (2) is applied using this $P_{3D_{\mathrm{composite}}}$​.

The fusion parameters $\left(w_{2D},w_{3D}\right)$ are critical for dynamically balancing the contributions of the 2D and 3D models. This ensures that the precise 3D contextual information from the refined subvolumes is leveraged where local ambiguities are high, while relying on the computationally efficient 2D segmentation in less complex or non‐uncertain regions. To determine their optimal values, the Particle Swarm Optimization (PSO) algorithm was employed.^38^, ^39^ In this framework, each particle represents a candidate solution defined by a vector $\left(w_{2D},w_{3D}\right)$, subject to the non‐negativity constraint $w_{2D},w_{3D}\geq 0$. The optimization objective was to maximize the Dice Similarity Coefficient (DSC) across the target tumor subregions (ET, TC, and WT).

#### 2.2.4 Model implementation and evaluation

The BraTS 2020 dataset was randomly partitioned into training and test sets using an 8:2 split ratio, resulting in 300 training cases and 69 test cases. The proposed uncertainty‐guided segmentation models were implemented independently for each of the three segmentation targets: ET, TC, and WT. Initially, three 2D nnU‐Net models with spherical projection were trained to generate 2D segmentation masks and associated uncertainty maps slice‐wise across the (MP‐MRI) input volumes. Note that the spherical projection origins can be limited to the intracranial volume (brain ROI) in the actual implementation, thereby eliminating redundant computations in non‐informative background regions. Subsequently, high‐uncertainty regions were extracted for each target separately using the kernel‐based analysis described in Section B.2. Three separate 3D nnU‐Net models were then trained using these extracted subvolumes to segment ET, TC, and WT kernels. All components of the proposed framework—including model training, identification of high‐uncertainty regions, development of 2D and 3D nnU‐Net models, and PSO algorithm execution—were confined to the training data. Training for both the 2D nnU‐Net and 3D nnU‐Net models was conducted using the PyTorch framework. A binary cross‐entropy (BCE) loss function was adopted, and optimization was performed using the Adam optimizer with an initial learning rate of $1\times 10^{-5}$. A batch size of 10 was employed, and training sessions were guided by early stopping criteria based on the validation DSC to prevent overfitting. The PSO was configured with a population size of 20 and run for 50 iterations to ensure convergence. The choice of BCE loss was motivated by the framework's reliance on uncertainty quantification via Shannon entropy, which requires the preservation of well‐calibrated voxel‐wise probability distributions. Other loss functions, for example, DSC‐based loss functions, often encourage overconfident predictions (pushing probabilities sharply to 0 or 1), which can artificially suppress the entropy signal necessary for identifying ambiguous regions. The alignment with the DSC evaluation metric is instead handled during the fusion stage, where the PSO algorithm explicitly optimizes the weights to maximize the global DSC.

Model performance was finally evaluated on the 69 independent test cases using four standard quantitative metrics: DSC, 95% Hausdorff Distance (HD95), voxel‐wise sensitivity, voxel‐wise specificity, and voxel‐wise accuracy. HD95 measures the maximum distance (at the 95th percentile) between the predicted and ground truth surfaces, and it is highly sensitive to boundary misalignment and shape correctness. These metrics were calculated separately for each target tumor subregion (ET, TC, and WT). Experiments were executed on a computational server equipped with AMD EPYC 7763 (64 cores) and NVIDIA L20 GPU (48G VRAM).

### 2.3 Comparison study

To rigorously assess the effectiveness of the proposed uncertainty‐guided segmentation framework, a series of comparative experiments were conducted against two benchmark models:

1. **2D nnU‐Net with spherical projection (without 3D refinement)**: This model evaluated the standalone performance of the spherical projection‐based segmentation approach. It accepted the original 4‐channel MP‐MRI slice as input and applied spherical projection to generate 1024 (with 8×8 projection center intervals) deformed versions of each slice. Each projected image was independently processed to produce segmentation probability maps, which were subsequently averaged to yield the final segmentation output. Similarly, three 2D nnU‐Net models with spherical projection were trained independently for ET, TC, and WT, and predictions were stacked across slices to reconstruct the volumetric output.
2. **3D nnU‐Net (fully volumetric model)**: A 3D nnU‐Net model served as a high‐capacity volumetric benchmark. Three separate models, accepting the full 3D MP‐MRI volume (with four modalities) as input, were trained to perform end‐to‐end volumetric segmentation for ET, TC, and WT, respectively.

For all comparative models, the training configurations were kept consistent with the proposed uncertainty‐guided framework, including training/test set partitions, the use of BCE loss, and the same optimizer and initial learning rate. The models were evaluated on the same independent test cases using DSC, voxel‐wise sensitivity, specificity, and accuracy. Statistical comparisons between the proposed uncertainty‐guided model and each comparison model were conducted using the Wilcoxon signed‐rank test. Since the proposed method was compared against two baselines (vs. 2D nnU‐Net and vs. 3D nnU‐Net), the Bonferroni correction was applied to adjust the significance threshold from 0.05 to 0.05/2 = 0.025.

## 3 RESULTS

**FIGURE 4:**
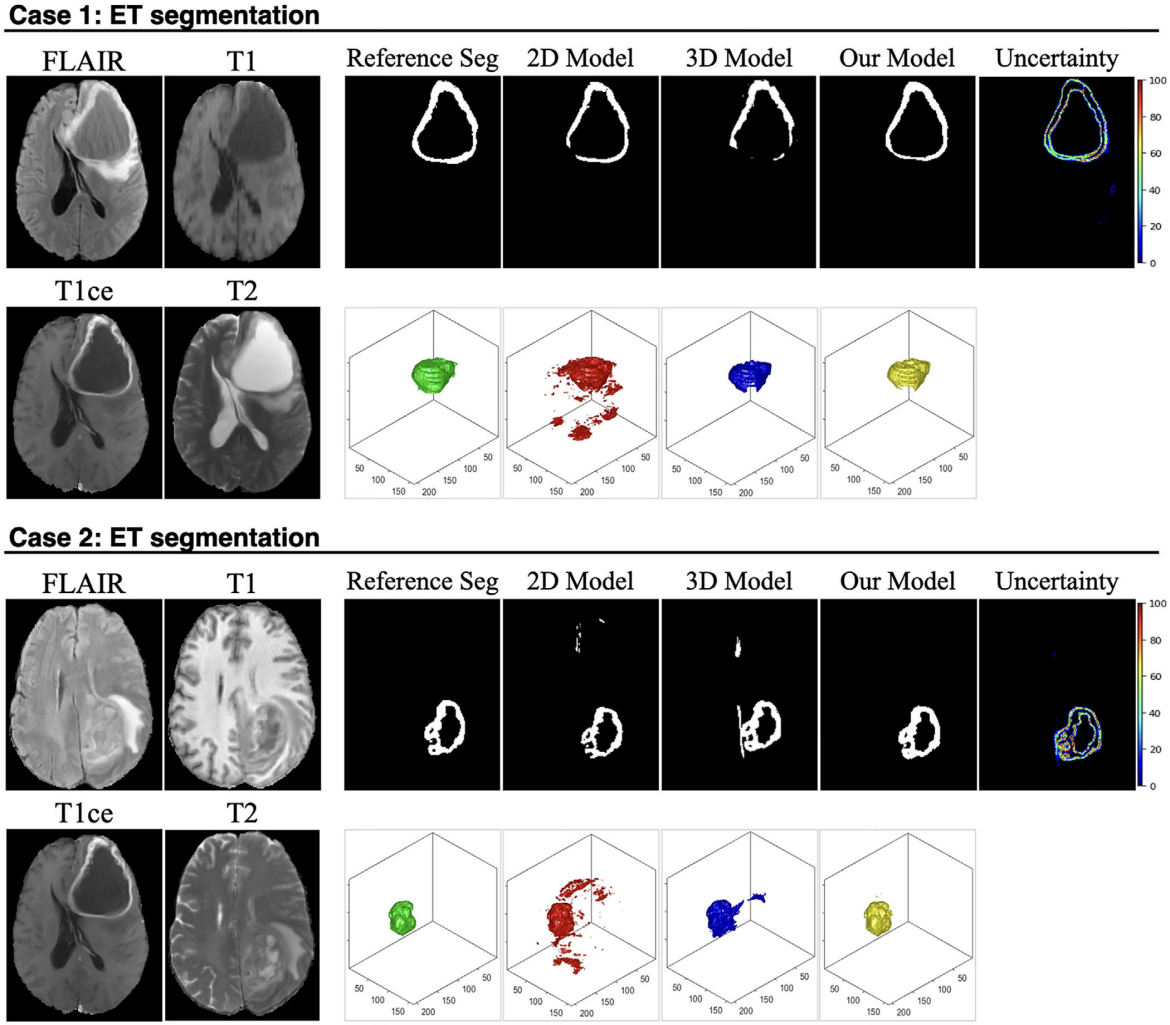
Visual comparison of ET segmentation performance across representative cases. For each case, the four input MRI modalities (FLAIR, T1, T1ce, and T2) are shown on the left. The first row of each case presents the 2D slice‐wise segmentation results, including the reference standard segmentation (reference seg), predictions from the 2D baseline model, the 3D baseline model, and the proposed model, followed by the voxel‐wise uncertainty map generated by the proposed framework. The second row illustrates the corresponding 3D reconstructions of the ET region for each method.

**FIGURE 5:**
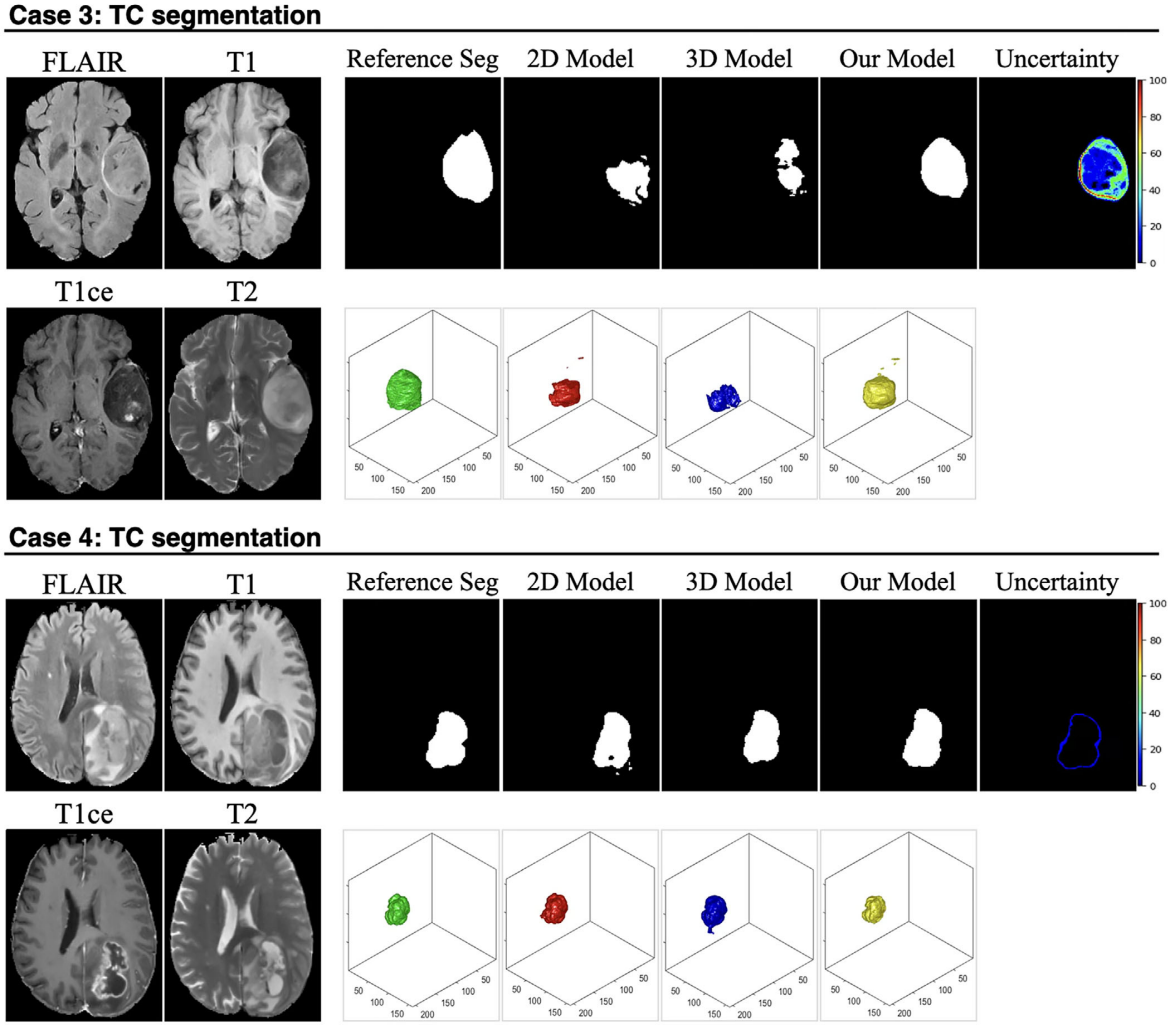
Visual comparison of TC segmentation performance across representative cases. For each case, the four input MRI modalities (FLAIR, T1, T1ce, and T2) are shown on the left. The first row of each case presents the 2D slice‐wise segmentation results, including the reference standard segmentation (reference seg), predictions from the 2D baseline model, the 3D baseline model, and the proposed model, followed by the voxel‐wise uncertainty map generated by the proposed framework. The second row illustrates the corresponding 3D reconstructions of the TC region for each method.

**FIGURE 6:**
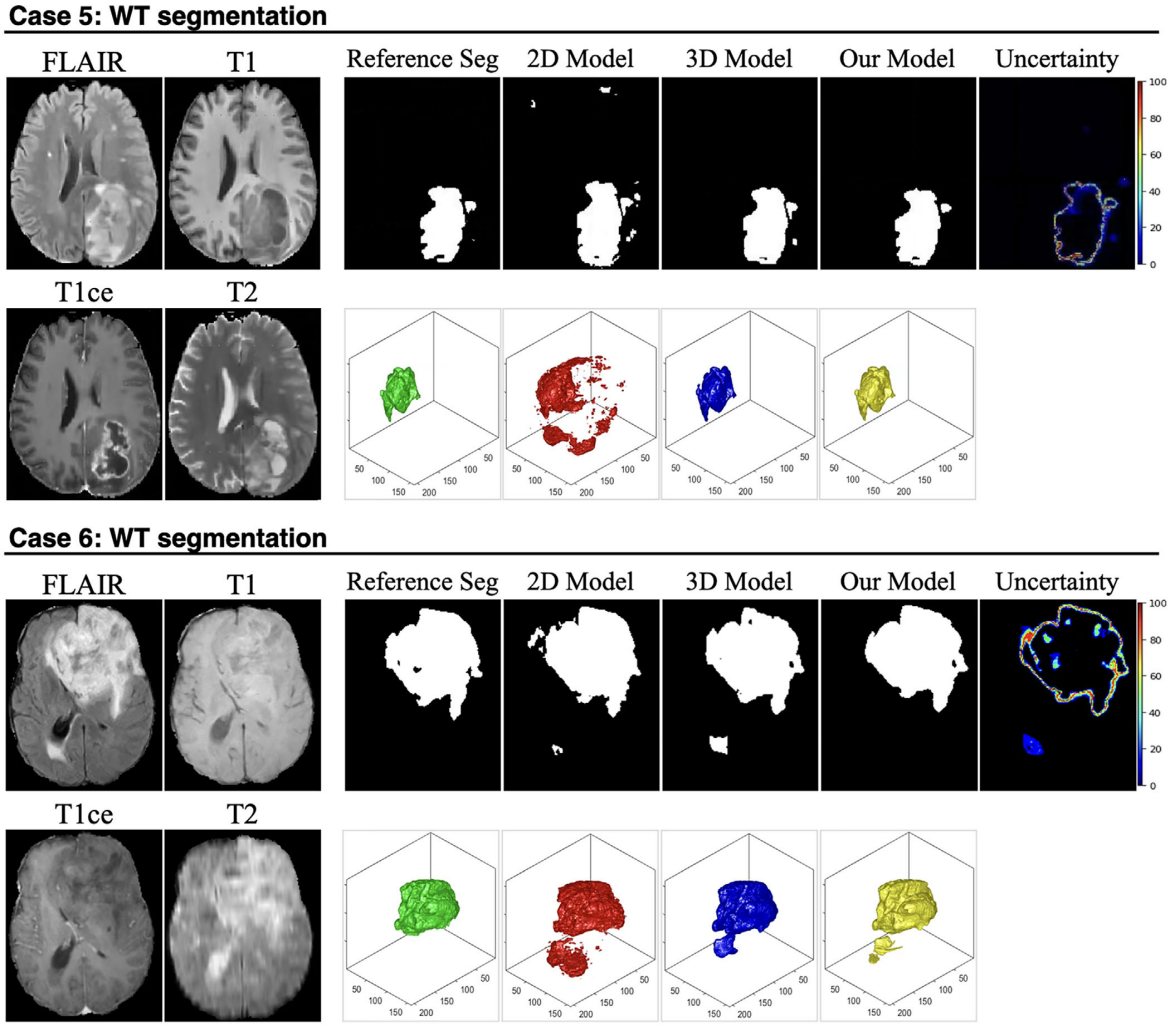
Visual comparison of WT segmentation performance across representative cases. For each case, the four input MRI modalities (FLAIR, T1, T1ce, and T2) are shown on the left. The first row of each case presents the 2D slice‐wise segmentation results, including the reference standard segmentation (reference seg), predictions from the 2D baseline model, the 3D baseline model, and the proposed model, followed by the voxel‐wise uncertainty map generated by the proposed framework. The second row illustrates the corresponding 3D reconstructions of the WT region for each method.

Figures 4, 5, 6 present representative examples from the test set, demonstrating the effectiveness of the proposed uncertainty‐guided refinement pipeline across multiple glioma subregions. To provide a comprehensive evaluation, we visualized the four MP‐MRI modalities (FLAIR, T1, T1ce, and T2), the reference standard segmentation, the predictions from the baseline models (2D and 3D nnU‐Net), the proposed uncertainty‐guided fusion model, and the corresponding uncertainty maps. Crucially, in addition to 2D axial slices, the 3D surface renderings of the segmented volumes were also provided. These 3D visualizations offer a distinct advantage in assessing the spatial consistency and topological integrity of the predictions, highlighting artifacts that are often invisible in single‐slice views.

- ET Segmentation (Figure 4, Cases 1 and 2): The limitations of the pure 2D model are most evident in the 3D renderings. In Case 1, while the 2D model appears to capture the lesion on the axial slice, its 3D reconstruction (red) reveals severe oversegmentation and spatial discontinuity, characterized by scattered “flying pixels” and fragmented artifacts lacking volumetric coherence. The pure 3D model (blue), conversely, tends to produce smoother but under‐segmented results, missing fine boundary details. The uncertainty map accurately highlights these ambiguous boundary regions. Guided by this uncertainty, our proposed model (yellow) successfully suppresses the discontinuous artifacts of the 2D model while preserving the detailed structure, resulting in a coherent volume that closely matches the reference standard (green). Similarly, in Case 2, the 2D model exhibits fragmented prediction islands, whereas our model recovers a unified lesion structure.
- TC Segmentation (Figure 5, Cases 3 and 4): In Case 3, the 2D model suffers from significant false‐positive predictions (oversegmentation) surrounding the main tumor mass, appearing as noisy debris in the 3D view. The 3D model provides a cleaner shape but lacks precision at the necrotic core boundaries. The uncertainty map effectively flags these heterogeneous regions as high‐uncertainty areas. Our model leverages this information to filter out the spurious 2D noise, yielding a compact and topologically accurate segmentation. In Case 4, where the core is embedded in edema, our model demonstrates superior specificity, avoiding the over‐inclusion of noncore tissue observed in the baseline predictions.
- WT Segmentation (Figure 6, Cases 5 and 6): In Case 5, the 2D baseline produces a jagged and irregular surface in the 3D rendering, reflecting inconsistent predictions across adjacent slices. Our model significantly improves surface smoothness and spatial integrity. In Case 6, which features a large, complex tumor morphology, the 2D model again fails to maintain connectivity.

Quantitative results (including DSC, HD95, sensitivity, specificity, and accuracy) and corresponding *p*‐value for all segmentation targets are summarized in Table 1. With the Bonferroni correction, all *p*‐values are now evaluated against this stricter threshold (*p *< 0.025), where the “*” marks the statistically significant difference. The proposed uncertainty‐guided fusion model consistently demonstrated superior performance across ET, TC, and WT subregions, particularly regarding segmentation accuracy (Dice) and boundary precision (HD95).

**TABLE 1:** Quantitative segmentation performance on BraTS 2020 test set (mean ± standard deviation).

		2D Model	3D Model	Ours	*P*‐value (Ours vs. 2D)	*P*‐value (Ours vs. 3D)
**ET**	**3D DSC**	0.7527 ± 0.3551	0.7530 ± 0.3340	0.8124 ± 0.3136	0.0085*	0.0081*
	**HD95 (mm)**	15.6221 ± 16.3994	25.4086 ± 31.5749	14.6044 ± 19.9408	0.0231*	0.0037*
	**Accuracy (%)**	0.9999 ± 0.0002	0.9991 ± 0.0010	0.9999 ± 0.0002	0.9689	0.0352
	**Sensitivity**	0.9201 ± 0.2163	0.9191 ± 0.3741	0.9297 ± 0.2108	0.0083*	<0.0001*
	**Specificity**	0.9999 ± 0.0002	0.9997 ± 0.0004	0.9999 ± 0.0001	0.4120	0.0121*
**TC**	**3D DSC**	0.7002 ± 0.3103	0.7027 ± 0.2844	0.7499 ± 0.2205	0.0173*	0.0215*
	**HD95 (mm)**	17.8802 ± 23.1222	15.9344 ± 23.9506	13.4676 ± 20.1152	0.0041*	0.0238*
	**Accuracy (%)**	0.9949 ± 0.0076	0.9957 ± 0.0067	0.9952 ± 0.0063	0.9713	0.4891
	**Sensitivity**	0.6481 ± 0.3384	0.6914 ± 0.3311	0.7700 ± 0.2779	<0.0001*	0.0068*
	**Specificity**	0.9996 ± 0.0005	0.9989 ± 0.0017	0.9983 ± 0.0022	0.1285	0.2938
**WT**	**3D DSC**	0.8989 ± 0.0915	0.9038 ± 0.0052	0.9055 ± 0.0648	0.0187*	0.0342
	**HD95 (mm)**	12.2421 ± 17.2201	9.8244 ± 15.0091	8.0988 ± 12.9516	0.0023*	0.0214*
	**Accuracy (%)**	0.9963 ± 0.0032	0.9963 ± 0.0025	0.9963 ± 0.0030	0.9745	0.4812
	**Sensitivity**	0.8704 ± 0.1353	0.9081 ± 0.0857	0.9017 ± 0.1082	0.0150*	0.0910
	**Specificity**	0.9989 ± 0.0013	0.9981 ± 0.0017	0.9982 ± 0.0019	0.1140	0.8970

*Note*: *P*‐values < 0.025 are marked with *.

In the ET subregion, the proposed method achieved a mean DSC of 0.8124 ± 0.3136, significantly outperforming both the 2D model (0.7527 ± 0.3551, *p *= 0.0085) and 3D model (0.7530 ± 0.3340, *p *< 0.0001). Crucially, the model demonstrated enhanced boundary delineation, yielding the lowest HD95 of 14.6044 mm, a statistically significant reduction compared to the 2D (*p* = 0.0231), and 3D (*p* = 0.0037) baselines. Sensitivity was also markedly improved to 0.9297 ± 0.2108, effectively overcoming the low sensitivity observed in the pure 3D model (0.9191), while maintaining high specificity (>0.999). For TC segmentation, our model yielded similar improvements, attaining the highest DSC of 0.7499 ± 0.2205. This improvement was statistically significant against both the 2D (*p* = 0.0173) and 3D (*p* = 0.0215) models. The framework also achieved the best boundary precision with an HD95 of 13.4676 mm, significantly lower than the 2D (17.8802 mm, *p* = 0.0041) and 3D (15.9344 mm, *p* = 0.0238) results. Furthermore, the model exhibited a superior ability to detect tumor core voxels, as evidenced by a sensitivity of 0.7700, which was significantly higher than both baselines (*p* < 0.01). In WT segmentation, the proposed model achieved the highest mean DSC of 0.9055 ± 0.0648. While this represented a statistically significant improvement over the 2D model (*p* = 0.0187), the difference compared to the 3D model did not reach the strict Bonferroni threshold (*p* = 0.0342). However, notably, the fusion model demonstrated significantly better boundary adherence than both baselines, achieving an HD95 of 8.0988 mm (vs. 2D: *p* = 0.0023; vs. 3D: *p* = 0.0214). This suggests that while the volumetric overlap for WT is comparable to the 3D benchmark, the proposed method provides more precise boundary definition.

The training and validation curves for our model were provided in *Figure* S–2 of the *Supplementary Material*. In terms of computational efficiency, training requirements varied by architecture: each target‐specific 2D nnU‐Net required approximately 48 h to converge, while the corresponding 3D nnU‐Net models demanded 48–72 h due to the complexity of volumetric patch‐based learning. Regarding inference speed, the proposed model demonstrated a total inference time less than 4 min per subject (comprising ∼3 min for 2D inference and ∼1 min for 3D refinement). In comparison, the 2D only nnU‐Net and 3D nnU‐Net models took <30 s for single patient inference.

## 4 DISCUSSION

In this study, we propose a hybrid segmentation framework that integrates spherical‐projection‐based uncertainty estimation with region‐specific 3D refinement. Unlike prior approaches that typically treat segmentation and uncertainty quantification as separate objectives, our framework transforms uncertainty from a post hoc evaluation metric into an active spatial attention mechanism. This strategy aligns with the emerging consensus that model confidence should serve as an informative prior to guide computational focus. Visual inspection of Figures 4, 5, 6 confirms that the generated uncertainty maps act as high‐fidelity detectors for segmentation failure. For instance, in Case 1, the uncertainty map precisely outlines the ring‐enhancing periphery, where the 2D model typically produces fragmented boundaries. Similarly, in Case 3, the map effectively captures the epistemic ambiguity arising from heterogeneous intratumoral intensities; the complex texture of the necrotic core triggers a dense uncertainty signal throughout the lesion. These examples validate that the uncertainty module effectively flags diagnostically challenging topologies. These high‐uncertainty regions are subsequently cropped from the original MP‐MRI data (FLAIR, T1, T1ce, and T2) and fed into the 3D model to revisit ambiguous structures with full volumetric context.

The achieved quantitative outcomes (Table 1) are comparable to, and in several aspects surpass, the leading benchmarks from the BraTS 2020 Challenge.^40^ As illustrated in Figures 4, 5, 6, the proposed method addresses the limitations inherent to single‐modality baselines: it mitigates the inter‐slice discontinuity often observed in pure 2D models (shown as scattered segmented pixels across the volume), while avoiding the tendency of pure 3D networks to over‐smooth fine boundaries. Consequently, our model achieves an optimal balance between spatial coherence and boundary precision with significantly higher DSC and lower HD95 metrics. Furthermore, specificity metrics are reported alongside sensitivity as explicit clinical safety indicators. Since false‐positive expansion of tumor contours in radiotherapy planning can inadvertently increase the radiation dose to healthy functional tissues, preventing over‐segmentation is critical. The high specificity maintained by our model (close to 1.0) suggests that the improved detection capability for ET, TC, and WT is achieved through precise anatomical delineation rather than indiscriminate boundary expansion.

In this study, the PSO‐based optimization method was applied due to its robustness in optimizing non‐differentiable, non‐convex objectives (e.g., the DSC) within a low‐dimensional weight space.^38^, ^39^, ^41^, ^42^, ^43^ The optimized PSO fusion weights (Table 2) reveal a distinct trade‐off between 2D accuracy and 3D contextual information. For ET and TC, the framework strongly favors the 2D backbone ($w_{2D}$ = 0.5848 > $w_{3D}$ = 0.4152 for ET and $w_{2D}$ = 0.6980 > $w_{3D}$ = 0.3011 for TC). This preference is driven by the specific mechanics of the spherical projection strategy. ET and TC regions are characterized by heterogeneous textures (e.g., necrosis) and highly irregular morphologies that are prone to local prediction noise. While the 3D model utilizes volumetric context, it performs a single inference; in contrast, the 2D model aggregates predictions from 1024 unique projection angles. This ensemble‐like mechanism statistically reduces prediction variance, making it exceptionally robust against the internal texture ambiguity of the necrotic core and the irregular fragmentation of the enhancing rim. Conversely, WT segmentation is dominated by the 3D model ($w_{2D}$ = 0.3204 < $w_{3D}$ = 0.6796). WT is defined largely by peritumoral edema, which presents as a large, diffuse volume with low textural complexity but weak, fuzzy boundaries. Typically, edema requires consistent spatial delineation along the *z*‐axis rather than textural denoising. The 2D model often fragments these diffuse boundaries, whereas the 3D model leverages volumetric continuity to regularize the fuzzy edges, ensuring a coherent 3D shape. Thus, for WT, the need for geometric continuity suppresses the need for multi‐view refinement.

**TABLE 2:** Optimized fusion weights for each tumor subregion.

	$w_{2D}$	$w_{3D}$
**ET**	0.5848	0.4152
**WT**	0.3204	0.6796
**TC**	0.6980	0.3020

Regarding potential clinical deployment, several factors favor the proposed architecture. Although the BraTS dataset used in this study is isotropic, clinical data is frequently anisotropic (e.g., thick‐slice protocols). In such scenarios, our model processes initial 2D segmentation images slice‐by‐slice and is therefore inherently immune to through‐plane resolution artifacts. Moreover, the PSO‐driven fusion provides an adaptive safety mechanism: in cases where extreme anisotropy might compromise the efficacy of the 3D refinement module, the optimization process allows the model to assign higher importance to the robust 2D predictions, thereby maintaining segmentation stability. In terms of computational efficiency, our model allocates the most computational resources to high‐uncertainty regions, making it theoretically more resource‐efficient than full‐volume 3D processing. However, the current implementation achieves an end‐to‐end inference time of under 4 min per subject. Analysis indicates that the computational bottleneck still lies in the spherical forward and backward projections; future optimizations utilizing parallel computing could further compress the overall inference time. Crucially, our approach focuses on the process of uncertainty‐based fusion and is not tied to a specific model structure. Since the fusion mechanism operates independently of the feature extraction backbone, replacing the underlying networks with more powerful models (e.g., nnU‐Net v2) would lead to further performance gains, although this upgrade might entail a trade‐off in computational cost.

Despite the demonstrated effectiveness, several technical limitations warrant further consideration. First, the current framework quantifies uncertainty using Shannon entropy derived from prediction variance across spherical projections. While this approach is computationally efficient and yields spatially meaningful maps, it may not fully capture both epistemic and aleatoric uncertainty.^31^ More expressive modeling strategies, such as deep ensembles or Bayesian neural networks, could offer better calibration and robustness. Second, the region selection mechanism remains heuristically defined. Although the kernel‐based uncertainty ranking algorithm effectively identified high‐priority regions for refinement in this study, its reliance on empirically chosen parameters—such as kernel size *d*, overlap ratio, and stopping thresholds—introduces tunable hyperparameters that may not transfer well across datasets or imaging conditions. Large kernels risk diluting uncertainty localization by including irrelevant voxels, whereas overly small kernels may fragment semantically coherent regions, impairing the contextual performance of the 3D model. A more adaptive strategy informed by uncertainty distribution statistics could yield a more principled and generalizable solution. Third, the framework was evaluated exclusively on the BraTS 2020 dataset. The generalizability of the spherical projection approach remains untested in other clinical settings or imaging modalities such as CT or PET. Finally, we acknowledge that the current evaluation focuses primarily on segmentation accuracy and boundary consistency, rather than downstream clinical tasks. In oncologic imaging, biomarkers such as metabolic tumor volume (MTV) and total lesion glycolysis (TLG) are critical for decision‐making.^44^, ^45^ Incorporating such task‐based evaluations within the current study is nontrivial, as reliable MTV and TLG analysis requires standardized SUV calibration and harmonized PET/MRI acquisition protocols, which are beyond the scope of the present retrospective dataset. Future work could therefore extend the framework to task‐based clinical evaluations, including uncertainty‐aware MTV and TLG estimation, to investigate how segmentation reliability propagates to clinically actionable biomarkers.

## 5 CONCLUSION

In this study, we developed an uncertainty‐guided segmentation framework that combines voxel‐wise uncertainty estimation with targeted 3D refinement to enhance the accuracy and reliability of glioma segmentation from (MP‐MRI) data. By leveraging a 2D nnU‐Net with spherical projection for initial segmentation and uncertainty quantification, and selectively applying 3D nnU‐Net refinement to high‐uncertainty regions identified via a kernel‐based ranking algorithm, our method effectively allocates computational resources to anatomically ambiguous or low‐confidence areas. This uncertainty‐driven refinement strategy presents a computationally efficient, clinically relevant approach that can be generalized to other imaging modalities and anatomical structures.

## CONFLICT OF INTEREST STATEMENT

The authors declare no conflicts of interest.

## Supporting information




**Supporting File**: mp70360‐sup‐0001‐SuppMat.pdf

## Data Availability

The dataset employed in this study is publicly available.
