# Supplementary material for: A voxel‐wise uncertainty‐guided framework for glioma segmentation using spherical projection‐based U‐Net and localized refinement
Source: Med Phys. 2026 Feb 27;53(3):e70360. doi: 10.1002/mp.70360 (PMC12947053; doi:10.1002/mp.70360)
Supplement: Supplementary file 1 — Supporting File: mp70360‐sup‐0001‐SuppMat.pdf [file MP-53-0-s001.pdf]

Supplementary Documents for the "*A Voxel-Wise Uncertainty-Guided Framework for Glioma Segmentation Using Spherical Projection-Based U-Net and Localized Refinement*"

1. Kernel Size Selection

Cumulative distribution of maximum bounding box dimensions for glioma subregions was shown in Figure S-1. The plots show the statistical distribution of tumor dimensions for (a) Enhancing Tumor (ET), (b) Tumor Core (TC), and (c) Whole Tumor (WT). Red markers and dotted lines: Indicate the median (50th percentile) maximal dimension for each subregion (approx. 43 mm for ET, 54 mm for TC, and 84 mm for WT). Solid vertical lines: Indicate the empirically selected compact kernel sizes (32 for ET/TC, 64 for WT). Dashed vertical lines: Indicate the next available standard power-of-2 dimensions (64 for ET/TC, 128 for WT).

We deliberately "rounded down" to the nearest standard power-of-2 dimensions (32 and 64) rather than scaling up (to 64 and 128) for the ease of calculation and computational efficiency. This "compact kernel" strategy forces the 3D refinement module to prioritize high-frequency local boundary features. The sliding window mechanism then compensates for the size difference, ensuring that larger anatomical structures are seamlessly reconstructed via patch aggregation.

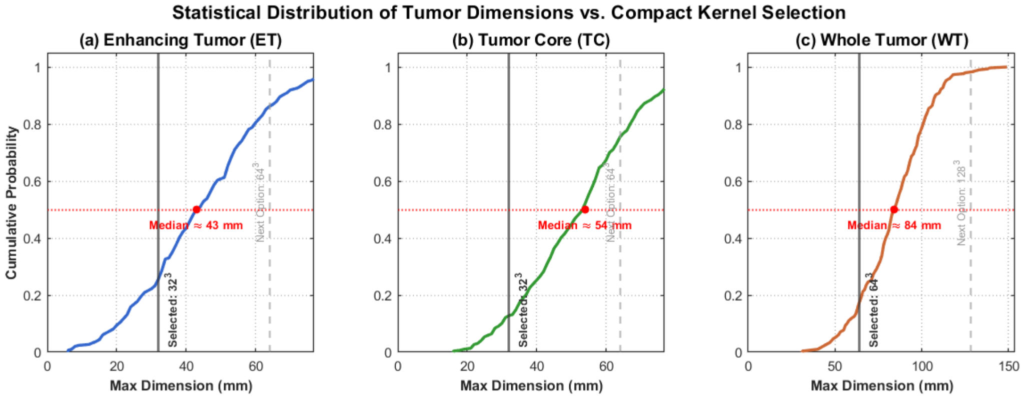

Figure S-1. Cumulative distribution of maximum bounding box dimensions for glioma subregions.

2. Training Curve

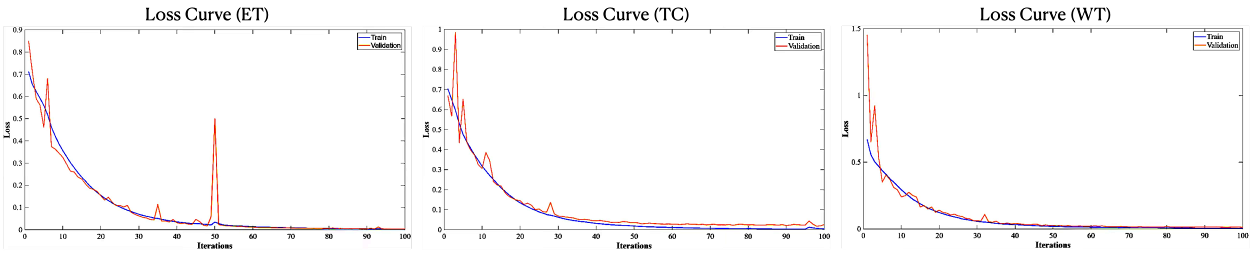

Figure S-2. The learning curves for our model for the segmentation of ET, TC, and WT, respectively.
